# Generators of Inequality and Inequity Affecting Dental Patient Safety: A Grounded Theory Approach

**DOI:** 10.3390/ijerph22081248

**Published:** 2025-08-09

**Authors:** Diego A. Gil-Alzate, Isabel C. Posada-Zapata, Andrés A. Agudelo-Suárez

**Affiliations:** 1 Faculty of Dentistry, University of Antioquia, Medellín 050010, Colombia; 2 National Faculty of Public Health, University of Antioquia, Medellín 050010, Colombia; isabel.posada@udea.edu.co

**Keywords:** patient safety, dental care, oral health, dentist-patient relations, qualitative Research, grounded theory

## Abstract

This study aimed to understand, through the voices of patients, the factors that contribute to inequality and inequity in oral healthcare and their implications for patient safety. A qualitative study was performed using a Grounded Theory approach (GT) through 13 in-depth interviews with a flexible design, recorded and transcribed verbatim for study purposes. Open and axial coding and analysis categories were generated, and a conceptual and explicative framework was established. Ethical approval was obtained. The main findings highlighted how individual, social, and contextual factors significantly influence the materialization of risks and failures in oral healthcare, ultimately affecting patient safety in dental practice. These factors include individual factors, the relationship between professionals and patients, and failures in healthcare service provision. Participants’ discourses showed examples of inequities, such as gender, socioeconomic gradient, educative level, type of healthcare system, discrimination, stigmatization, and othering-otherness, and their effect on dental care and dentistry safety. Health inequities should be tackled in a preventive and proactive manner through the effective integration of intersectoral policies and strategies. This approach would enhance oral health, make patient safety a fundamental pillar of dental care, uphold human dignity, and strengthen trust in the healthcare system.

## 1. Introduction

The universal access and coverage that implies for all individuals and communities to use health services and other structuring elements without discrimination, ensures quality and reliability [1,2]. However, identifiable gaps exist concerning individual, family, and community needs [3], particularly in light of growing inequalities and inequities in society, which inevitably affect safety in oral healthcare processes.

Health inequalities arise from the significant variation among individuals and groups in experiencing health-related events. While some differences may be due to chance, systematically different outcomes can be observed for groups with common characteristics [4]. Inequities refer to unjust, unnecessary, and avoidable differences in health outcomes, both within and between countries [5]. Furthermore, inequity does not only refer to injustice in distribution and access but also to the intrinsic processes that generate it [6]. For instance, individuals with lower socioeconomic status face a greater risk of poor health outcomes, which in turn increases the likelihood of processes that impact healthcare delivery and compromise patient safety [7].

Patient safety is defined as “*… the reduction of the risk of unnecessary harm associated with healthcare to an acceptable minimum, which refers to the collective notions of current knowledge, available resources, and the context in which care was provided, weighed against the risk of not providing treatment or providing another*” [8]. It is influenced by various factors, including the quality of healthcare, resource availability, the skills and knowledge of service providers [9], and social determinants of health. According to the World Health Organization (WHO), patient harm ranks 14th in contributing to the global burden of disease, with most of this burden falling on low- and middle-income countries, resulting in lost productivity and diminished trust in the healthcare system [10]. This underscores the impact of inequalities in ‘supposedly’ good quality and safe healthcare processes, revealing that the risk of harm during healthcare is experienced unequally, exacerbating existing vulnerabilities to poor health outcomes, particularly for those from socioeconomically disadvantaged, culturally, and linguistically diverse backgrounds [11,12,13,14,15].

Oral health, historically excluded from public health priorities, must be integrated into universal health coverage, as emphasized by WHO’s Global Strategy on Oral Health, which highlights equity and patient safety as core components [16,17]. Poor oral health outcomes, particularly among marginalized populations, are often mediated by access barriers, fragmented care, and undifferentiated clinical practices. These conditions increase the likelihood of adverse events and unsafe care experiences, reinforcing a cycle of clinical and structural vulnerability [14,15]. While health inequalities and inequities have been examined in various reports in Colombia [18,19,20,21], there is still a noticeable gap when it comes to understanding how these issues impact patient safety in oral healthcare, especially from the viewpoint of patients in Colombia. This is important to consider, as national and local research has identified certain barriers to accessing oral health services across different population groups and social contexts [22,23,24,25]. Another specific study conducted in workers with subsistence jobs from Medellín (the second largest city in Colombia) showed a poor oral health perception, and this situation is associated with barriers to oral healthcare [26].

The scientific literature highlights a substantial body of epidemiological studies aimed at characterizing the prevalence of adverse events in dental practice within public and private institutions, providing a statistical overview across various general and specialized dental care services [27,28,29,30]; the same situation is observed in Colombia [11,31,32]. These studies have documented factors related to communication failures, diagnostic omissions, and unequal treatment, all of which increase the risk of unsafe care. However, there is a pressing need to advance knowledge on the factors affecting the safety of dental practice, considering the perspectives of various stakeholders and employing diverse conceptual and methodological approaches [33]. The relationship between oral health inequity, patient safety, and dental practice has not been sufficiently explored.

Qualitative research enables the exploration of the social construction of dental practice and patient safety by examining the perceptions, opinions, experiences, and perspectives of those directly involved in this social phenomenon [34]. This research perspective has gained increasing prominence in dental research [35]. There are different methodological approaches, and one of them is grounded theory (GT). Conducting a study using a GT approach involves certain ethical considerations that justify its implementation, such as respect for human dignity as a fundamental right, the recognition of oral health as an integral component of the basic right to health, the legitimization and amplification of the voices of individuals who experience the care process, and the unveiling of aspects not captured by quantitative and epidemiological indicators.

Accordingly, this study aimed to understand, through the voices of patients, the factors that contribute to inequality and inequity in oral healthcare and their implications for patient safety.

## 2. Methods

### 2.1. Context, Approach and Design

This study was conducted in Medellín, the second largest city in Colombia, in urban settings with a mix of public and private dental services. The city covers an area of 376.4 square kilometers and has an estimated population of 2.6 million inhabitants, with a higher proportion of women (53%). It has good coverage of public services —such as potable water, sewage, electricity, and waste collection—although coverage is lower in rural areas. In recent years, the population structure has changed significantly, with an increase in the proportion of older adults (aged 65 and over). Regarding economic and quality-of-life indicators, the unemployment rate has been decreasing over the past five years and is currently below 10%. However, significant social and quality-of-life inequalities and inequities persist across different areas of the city [36].

Given the scope, research objectives, and participating subjects, an inductive, interpretative, and emergent qualitative study was proposed [37], utilizing a Grounded Theory (GT) perspective as the methodological strategy [38,39,40,41,42]. Grounded Theory (GT) is a qualitative methodological approach that allows the generation of theoretical constructs based on empirical data, particularly when the subject of study is insufficiently conceptualized or lacks theoretical models [38,39,40,41,42]. GT enables the identification of relationships, patterns, and underlying processes in social phenomena by coding and categorizing information from participants’ experiences. From this conceptual and methodological approach, GT grants epistemic prominence to patients, decentralizes the traditional biomedical view, and generates knowledge that can influence public policy, professional training, and the improvement of healthcare delivery. Moreover, GT emphasizes participants’ voices, facilitating the construction of knowledge from their perspective. The lead author, a dentist, engaged in critical reflection on her professional role throughout data collection, fostering a dialogical and horizontal researcher–participant relationship. Establishing this kind of relationship in qualitative research involves fostering an equitable and respectful interaction between the researcher and the participants, recognizing their agency, knowledge, and experiences as valid and essential.

### 2.2. Participants

The selection process for participants was based on specific and theoretical criteria regarding their ability to provide detailed insights into the research topic. Specifically, we chose individuals who had experience as dental patients and who could represent different forms of inequality, such as differences in gender, ethnicity, migration background, socioeconomic status, and place of origin; in this case, men and women over 18 years of age, dental patients, and residents of Medellín and/or the Metropolitan Area. Purposive and convenience sampling strategies were employed for participant selection, utilizing various recruitment mechanisms defined by the research group, including community contacts, service announcements, social media, and the snowball method or nominated sampling, through people referred by the study participants [43]. The process of selecting participants was flexible and ongoing; as we analyzed the data, our criteria for inclusion evolved to better capture emerging categories. The determined sample size was defined by theoretical saturation, confirming in the 13th interview that no new data or categories emerged relevant to the study objectives [37]. Table 1 provides a general sociodemographic overview of the 13 interviewees.

### 2.3. Fieldwork and Data Collection Techniques

Fieldwork was carried out between September 2023 and June 2024, consisting of 13 in-depth interviews with a flexible design to adapt to participants’ responses and contributions. The analysis considered a group of themes and axes previously defined by the research group (Table 2). The interview process was a free dialogic exchange, conducted without pressure or harassment, which facilitated interaction between the interviewer and interviewee and allowed for the generation of new questions or emerging categories. Each interview included a sociodemographic form. Although an interview script was utilized (Supplementary File: Annex 1), flexibility was permitted to adapt to unexpected responses and contributions from participants.

All interviews were conducted by the main researcher (D.A.G.-A.), a male dentist with postgraduate studies in Patient Safety and Healthcare Quality. This process was supervised by other members of the research team. The first supervisor (I.C.P.-Z.) is a female psychologist with a PhD in Social Sciences and expertise in qualitative methods (GT). The second supervisor (A.A.A.-S.) is a male dentist and Psychologist with a PhD in Public Health and expertise in topics related to oral health inequalities and inequities and qualitative methods.

Fieldwork was complemented by observation notes from the main author and research team meetings to review aspects related to methodology and the analysis plan. The interviews were recorded and transcribed verbatim.

### 2.4. Data Analysis

**Figure 1:**
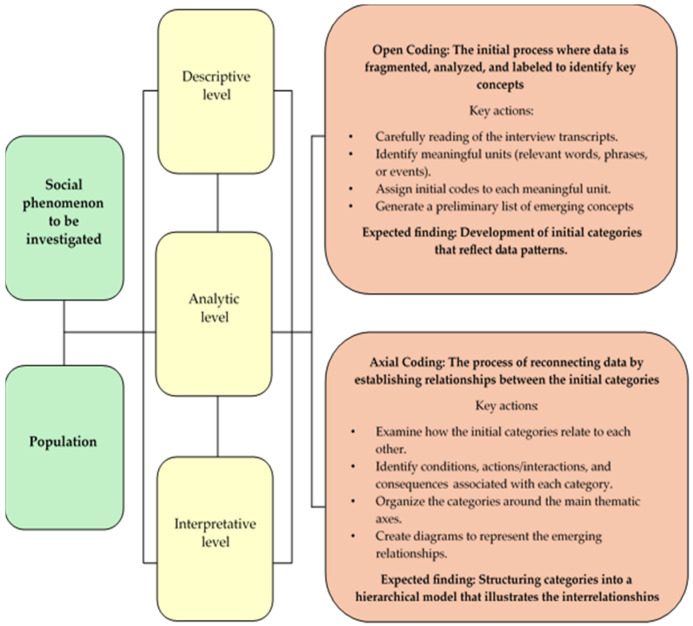
Phase outline for the analysis plan.

An initial reading of the data was conducted, and segments were manually identified, highlighting significant fragments, attributing meanings, and identifying codes. The coding process was carried out by the first author (D.A.G.-A.), who received ongoing supervision from the second author (I.C.P.-Z.). A process of reflexivity and analysis of the implications of the meanings of the codes and their corresponding keywords was conducted subsequently. Further discussions with the third member of the research team were carried out (A.A.A.-Z.), providing clarification on specific thematic aspects related to patient safety, oral health inequities, and inequalities. Codes represent fragments deemed valuable for the research, which were later grouped into categories corresponding to thematic axes identified through theoretical data saturation [38,39,40,41,42]. These categories were defined through a consensus between the two coding authors (D.A.G.-A., I.C.P.-Z.). During open coding, analysis categories were generated, grouping a series of codes according to their thematic content. Finally, in the axial coding stage, the descriptive categories that initially emerged were refined into axial categories validated by a methodological expert as a strategy to ensure the rigor of the process (I.C.P.-Z.), as illustrated in Figure 1.

The interview process, aimed at achieving a more robust analysis, was carried out in two phases. Specifically, stage 1 of the process involved the implementation of six interviews, from which 256 codes emerged through microanalysis, leading to 37 open categories. After analysis, these were consolidated into seven descriptive categories (Table 3). Stage two was conducted with seven interviews, in which descriptive categories were explored in depth, and a second axial coding was performed. This step allowed for the development of a summary conceptual framework that establishes the relationships between the analysis categories. It is important to note that, in terms of methodological rigor, this study employed tools only up to the axial coding phase, and no selective coding was performed.

Transcripts were made available to participants who expressed interest in reviewing and validating their content. This practice served as both an ethical and methodological validation strategy, allowing feedback on the main categories and codes. No specific software was utilized for the qualitative data analysis process, and alternative tools such as Microsoft Word and Excel for Windows were considered instead. The results present verbatim excerpts from participants’ discourses that best represent the analyzed topics, identifying the interview number and the participant’s sex.

### 2.5. Ethics

Participation was voluntary, and oral and written informed consent was obtained. Confidentiality was maintained throughout the research process in accordance with Colombian regulations (Resolution No. 008430/1993—Ministry of Health and Social Protection) [45]. This study adhered to international standards for human research (Declaration of Helsinki and International Ethical Guidelines of the Council for International Organizations of Medical Sciences in collaboration with the World Health Organization [46,47]. Given the qualitative nature of this research, interviews were handled appropriately, and the privacy and potential vulnerability of participants were always respected. This study is considered risk-free and was approved by the Bioethics Research Committee of the Faculty of Dentistry of the University of Antioquia (Act 09/2023, Concept Nº 164).

## 3. Results

The main findings from the interviews provided insight into how, from the perspective of dental patients affected by factors generating inequities and inequalities, there is a clear risk that failures in the care process could spark adverse events against them. These results are consistent with the methodological design, following the descriptive and analytical phases of the study and considering the initial open and axial coding processes.

Overall, participants shared not only their personal experiences and perspectives but also recounted situations lived by people close to them, often integrating these narratives as their own. Such accounts are considered valid and meaningful within the framework of the qualitative methodology employed, as they reflect the social and emotional realities that shape participants’ understanding of the phenomenon under study.

**Figure 2:**
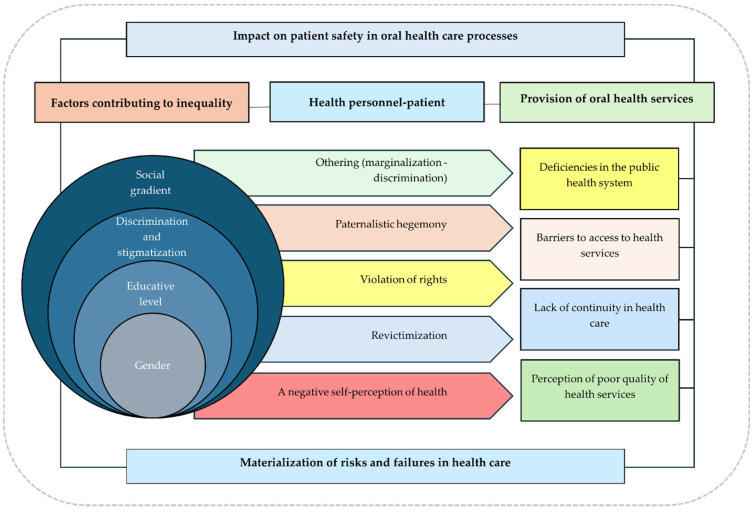
Conceptual framework for understanding the relationship between the impact on patient safety and the factors that generate inequity and inequality.

Figure 2 illustrates a conceptual framework depicting the primary relationships between the categories of analysis identified in the participants’ discourse, considering trends, agreements, and disagreements in the reported information. This figure explains how individual, social, and contextual factors significantly influence the materialization of risks and failures in oral healthcare, ultimately affecting patient safety in dental practice.

In this context, a lower payment capacity increases the likelihood of access barriers, worsening health conditions, which undermines the right to quality healthcare, exacerbating patient suffering and leading to harm. A critical category that emerges from this is “othering, [48,49]”, highlighting dynamics of marginalization and discrimination that significantly impact patient safety. This issue is closely tied to professional–patient interactions, in which some professionals exert excessive control over patient decisions, violating their autonomy and further victimizing individuals who have already faced discrimination. This perpetuates a cycle of neglect and harms their safety.

The following subsections will summarize the most important aspects from the discourse of the study participants, explaining key topics and general themes.

### 3.1. Socioeconomic Gradient

For most interviewees, socioeconomic factors determine their lack of access to dental services perceived as safe and of higher quality. This factor is directly proportional to the risk of failures and adverse events. In other words, the lower their ability to pay, the lower the quality of care they receive, and consequently, the higher the risk of experiencing harm during treatment:

*[…] Look, I’ll be honest with you, when it comes to healthcare, especially dentistry, those with money are the ones in control. Doctors treat those who can pay better—they give them more time and do higher-quality work… We, the poor, have to settle for the leftovers. There are never any appointments; they discriminate against us for smelling bad, and sometimes they don’t even treat us at all. It’s like we’re ‘worth nothing (Una mierda, in Spanish).*

(Int8, woman)

This aspect emerged as a critical determinant, with participants highlighting the lack of access to quality dental services due to poverty, as well as the discrimination and stigmatization they experience because of this condition.

### 3.2. Gender

Gender emerges as an analytical category that transcends its representation as a dichotomous variable. It should be understood through cultural representations and social and historical constructions in a non-binary manner [50]. In this study, it is also linked to the concepts of masculinity and femininity and their relationship with dental care processes. Regarding situations of inequality and inequity based on sex/gender, the interviews revealed perceived preferential treatment based on physical appearance and personal presentation. Participants highlighted feelings of racism and classism in healthcare provided by some professionals, leading to perceptions of discrimination and unequal treatment. Additionally, age was also a factor, as older adult women reported different or less careful treatment compared to younger women:

*[…] A 68-year-old lady […] was treated very poorly. She had a dental prosthesis and had an issue with it, so she went in. Look, she wasn’t there for even five minutes, and they didn’t do anything for her, doctor. […] I don’t know exactly who she saw because there are several dentists there. It’s a big room with multiple cubicles, and she walked out crying.*

(Int2, woman)

On the other hand, healthcare provided by female professionals (specifically in the case of female patients) was identified as generating greater comfort and trust. In this sense, care by female professionals was perceived as warmer and more empathetic in interactions. Although subtle, there were noticeable differences in the care received from male dentists, with kindness and sensitivity being associated with women, while men were linked to technical skills and roughness. These perceptions reflect deeply rooted gender stereotypes that influence patient–professional interactions:

*Yes, of course, female doctors treat patients better; they are kinder. They explain things to calm your nerves and treat you with more kindness. I remember that when I was in pain, it was a young woman who helped me so that it wouldn’t hurt again—she was very sweet. Men, on the other hand, are sometimes rougher, more distant, like you’re wasting their time.*

(Int8, woman)

*So yes, I do think there is a difference in the way women provide care in the field of dentistry. When I was treated by a male dentist at some point, I always felt a bit of roughness—let’s say, a certain level of forcefulness. Well, we’re men, and as men, we tend to have more strength than women.*

(Int6, man)

### 3.3. Education Level

According to the interviewees, access to dental services and the quality of care received varied significantly based on factors such as geographic location, as well as educational level and type. Additionally, knowledgeable and well-informed patients were more likely to assert their rights and demand respectful treatment. This awareness reduced their vulnerability to the concealment of errors in the care process:

*Vulnerable, completely, yes. Regardless of their age, sexual orientation, socioeconomic status, race, religion, or anything else, I believe that a patient who lacks knowledge is entirely vulnerable […]*

(Int4, man)

Moreover, patients reported that some professionals disregarded protocols in situations where they perceived a lack of knowledge and education on the part of the patient. This induced certain errors in their care. They also suggested that professionals tended to rush through treatment when they sensed that the patient did not fully understand the procedure:

*And that’s another issue—people think that medical staff are doing them a favor. No, they are workers. That doesn’t mean they should be treated badly, of course not, but it does mean we should remind them: ‘Hey, you have a job to do, and this is your responsibility.’ So, don’t put the burden of your job on me. I think, overall, the system works well—but it works well for people like me, who have knowledge and a clear understanding of how it functions. But in general, most people don’t have that information, which is why navigating the system becomes so complicated.*

(Int5, man)

### 3.4. Discrimination and Stigmatization

In several patient narratives, it became evident that differences in healthcare services exist based on individuals’ vulnerability factors. For example, being a woman or having a darker skin tone led to experiences of racial discrimination in dental care, contributing to a perceived disparity in treatment quality. Additionally, some participants reported that patients’ medical histories served as a barrier to receiving proper care, as discrimination and a lack of sensitivity within the healthcare system were particularly evident toward LGBTQIA+ individuals:

*[…] In the case of HIV specifically, many people abandon their treatments because they don’t want to return to a place where they felt judged, mistreated, and told how they should live their lives.*

(Int5, man)

*I have experienced being treated with disgust, being made to wait for a long time, and being told that my problem wasn’t important… And the worst part is that, because of those bad attitudes, you end up in pain, with more damage, or with treatments that weren’t done properly. It’s as if you don’t have the right to good care just because you don’t have money.*

(Int8, woman)

### 3.5. Othering

From this perspective and based on analyzing and interpreting the collected data, an emerging category was identified: distrust in the dental care system, stemming from perceived aspects of poor quality and insecurity, deeply linked to discrimination and marginalization. A predominant element in the participants’ perceptions, connected to the previously described issues, was othering—a largely invisible category in their interactions but one that became evident through their experiences of exclusion, stigmatization, and a lack of humanization in healthcare services [48,49]:

*At some point, they extracted the wrong tooth from my cousin, and it took them a long time to fix the damage. My cousin lives in a small town, and I don’t know if it was because she’s a woman, because she lives in a rural area, or because she was earning minimum wage—but for some reason, they didn’t want to resolve her issue promptly. And I do believe that in dental care, things can happen that end up harming patients.*

(Int6, man)

*You have no rights when you’re poor. Plus, I’m Black and come from far away, and if you don’t allow yourself to be mistreated, they see you as rude and call you “los tombos” (a Colombian term referring to policemen). If you complain or demand proper care, you’re afraid of retaliation or being treated even worse if you come back to insist.*

(Int8, woman)

*It’s terrible when a doctor has no compassion for you. If they don’t understand what you’re going through, if they don’t put themselves in your shoes, how can they treat you well? I’ve experienced being kicked out of a clinic by a very rough doctor—after waiting for hours, he told me there was no more time to see me, and I was in so much pain. But I heard him say that it was disgusting to treat me… or they’ve told me I’m exaggerating my pain. It’s as if I weren’t even a person.*

(Int8, woman)

### 3.6. Relationship Between the Health Professional and the Patient

According to the interviewees’ discourses, a poor interpersonal relationship between the professional and the patient can lead to failures in care that directly impact the quality and safety of treatment. Additionally, it fosters distrust in the patient regarding the technical and scientific competence of the professional:

*There are doctors who earn their degree just for the sake of having it. There’s a real lack of human compassion—that’s what I’ve noticed in my appointments. Today’s doctors lack empathy… they’re only after what they can earn and a title.*

(Int2, woman)

Participants noted that the lack of empathy in care and the failure to recognize the patient as a person were key factors that diminished their trust and satisfaction in their relationship with the professional:

*Some doctors think they’re gods. They feel all-powerful and treat you as if you’re less than them. They discriminate against you because of how you smell, give you appointments whenever they feel like it, make you wait for hours, or don’t see you at all and kick you out of the hospital… You have to put up with it because if you don’t, they’ll never treat you.*

(Int8, woman)

### 3.7. Healthcare and Oral Healthcare Models

The factors that generate inequality and inequity at both the individual and social levels, as previously mentioned, are closely linked to dental health services and, consequently, to patient safety and dental practice. This is related to the adequate provision of health services, which is in turn closely tied to the Colombian General System of Social Health Security (SGSSS acronym in Spanish). Deficiencies within the health system are evident, reflected in access barriers, lack of continuity in dental care, and a perceived low quality of health services. All of these issues are connected to the existing models of oral healthcare delivery.

In this sense, interviewees observed a significant difference between private and public healthcare models, leading to variations in the quality of care. The primary issue in the public sector was the extremely short consultation times, which significantly increased the risk of errors due to a lack of continuity, prolonged waiting times, frequent appointment cancelations, and unnecessary treatment interruptions:

*I went to get a filling… I had a toothache, and they told me I needed a filling, so I agreed. But after they did it, there was still a hole, and I felt a cold sensation. I kept wondering why, so I went back, and they told me it was normal. Then, I scheduled an appointment at a private clinic […] and it turned out they had really left a hole. So, the dentist had to perform […] a minor surgery, they even had to put in stitches, and then they fixed it… Well, at least I didn’t lose the tooth.*

(Int2, woman)

*[…] Every day on the news and specifically in my family, I’ve heard relatives talk—especially those who go to public dental care—about the difficulties they’ve had, mainly when trying to book an appointment. And when it comes to specialists, it’s even worse.*

(Int6, man)

## 4. Discussion

### 4.1. Summary of Study Findings

In the context of this research, GT enabled an in-depth understanding of how structural and relational factors contribute to the generation of inequalities and inequities that impact patient safety in dental care. Main findings highlighted that experiences of people undergoing dental care influence these factors. However, contextual elements—such as geographic, social, economic, and political factors—also play a role, as they are closely linked to the healthcare system. Similarly, this study addresses patient safety from an experiential perspective, revealing how structural and relational conditions shape patients’ perceived risks in dental care. To the best of our knowledge, this study in Colombia is the first to qualitatively explore topics related to dental patient safety using GT.

### 4.2. Possible Explanations for the Study Findings

The results of this research provide evidence that oral healthcare could be unsafe, especially when there is a possibility of marginalizing and discriminating against patients. From a political point of view, the implementation of various neoliberal policies and ongoing social disputes has deepened inequities and generated even more avoidable inequalities is undeniable [51]. These include fragmented, disjointed, and uncoordinated oral healthcare services. Consequently, this has deteriorated people’s oral health status, and, ultimately, caused harm or injuries to patients. Although these harms may be unintentional, they arise due to a lack of access, timeliness, continuity, and, most importantly, safety.

This study contributed to establishing the relationship between the impact on patient safety and the factors that generate inequity and inequality in oral healthcare through a conceptual framework developed according to the principles of grounded theory. In recent years, there has been increased awareness of oral health inequalities observed between different social groups or within the structure of these groups as shown in several systematic reviews [52,53,54,55]. These studies highlight the individual, social, cultural, and contextual factors involved in the patterns of access to and use of oral health services across different populations and age groups [52,53,54,55]. This awareness has led to a better understanding that inequality is systemic and rooted in various socioeconomic and political structures [56]. The findings of this research highlight the need to examine how the root of current inequities lies within societal structures, including the distribution of power, money, and resources. However, there are individuals with greater vulnerability who exhibit worse oral health indicators. These inequities and inequalities themselves represent an increased risk for unsafe healthcare processes [15].

Gender emerged in the participant discourses as an analytical category to explain differences between men and women. The scientific literature about the topic indicate that men often have poorer oral health indicators, while women maintain better hygiene habits and seek dental care more frequently [57,58]. This could reflect a greater participation of women in research studies, as seen in a study conducted in the city of Medellín, where two-thirds of the participants were female. However, the study findings point out a contextual analysis that considers the intersectionality of gender with social class, ethnicity, country of origin, and education level. These factors contribute to inequities in healthcare access, as highlighted in this study. Tudor Hart’s concept of the “inverse care law,” introduced in the 1970s, underscores how populations with greater healthcare needs receive the least care [59]. Research conducted in high-income countries emphasizes this situation in vulnerable groups and contributes to explaining the qualitative findings of the present study [60,61,62].

Participants discourses and opinions related to the safety of dental practice encompass the relationship established with the oral health professional. These include stereotypes about the professional’s characteristics, scientific and technical competence, and empathy. Additionally, personal opinions about male and female dental professionals shape these perceptions. This is a key element in studying the use of oral health services, as the professional–patient relationship ensures the success of dental treatment and promotes adherence under conditions of quality, effectiveness, and safety. A qualitative study in Libya shows that factors influencing communication between patients and dentists include trust, information interactions, moral support, engaged involvement, emotional support, clear communication about procedures, and the dentist’s understanding of the patient’s emotions [63]. These results are complemented by another qualitative study conducted in Germany that expands research on patients’ decision-making regarding dental treatments [64]. This study highlighted categories that influence patient behavior, such as professional and social skills, and the relationship of trust between patient and dentist. Both studies are very comparable to the context of the city of Medellín.

Health services are considered an intermediate social determinant that impacts inequalities in health indicators and represent a contextual factor highlighted in the findings of the present study from two elements of importance. On the one hand, some factors affect or limit access to dental healthcare services, such as costs and insurance or healthcare system coverage. Limited insurance coverage for oral health and the targeting of public services constrain timely and continuous access, resulting in marked differences in perceived safety. On the other hand, there are conditions inherent to the dental service itself, which involve human resources, the professional’s technical skills and expertise, the trust and empathy generated during the care process, and the guidance the patient receives regarding treatment, among other factors. These results align with the findings of a scoping review aimed at mapping the determinants of access to dental services within a universal health coverage (UHC) framework in oral health [65].

These findings are highly relevant to the Medellín study. They expand the understanding that communication between the patient and the professional may involve factors that create inequality and inequity, ultimately impacting the safety and satisfaction of dental treatment.

Complementing the above, one of the theoretical categories that emerged strongly in the opinions and perspectives analyzed in the participants’ discourses, and which underscores the need to consider it intertwined in the discursive analysis of inequalities and inequities and their impact on health, is the concept of “othering.” This element evidently helps to understand the dynamics of social relationships, particularly in the context of healthcare. It relates to processes in which certain individuals or groups are perceived as negatively different, often leading to marginalization and discrimination [66]. This phenomenon has significant implications for health inequalities and inequities, particularly in dentistry, in which socioeconomic status, cultural context, and systemic barriers can influence access to care and, consequently, treatment outcomes [48,49]. Recognizing these factors is essential for developing an inclusive dental practice that addresses the needs of vulnerable, diverse populations while promoting equitable access to oral health services and ensuring safety.

While existing studies have characterized the problem from an epidemiological perspective [27,28,29,30], a deeper understanding is needed of both individual and contextual factors that may influence dental care and patient safety—from not only the patient’s standpoint but also the professional’s perspective. In this regard, two Colombian studies are particularly relevant. The first, carried out in 10 dental schools nationwide, monitored unsafe dental care practices (including adverse events, incidents, and healthcare failures) between 2015 and 2016 [11]. The second was a complementary, more specific study in a public educational institution in Medellín, which trains dentists [32]. Both studies highlight that unsafe dental care can be influenced by various factors, including the type of institution, the person reporting or experiencing the harm, and the sociodemographic characteristics of the patient receiving care. These results complement those reported in the qualitative study, which, through the conceptual framework of relationships between categories, expands the information on factors affecting patient safety from an equity-based perspective that identifies the potential axes of inequality in oral healthcare. Taken together, these three studies provide both an institutional perspective and the patient’s viewpoint within the dental care process.

### 4.3. Strength and Weakness of This Study

In interpreting the results, understanding the scope of the research in conceptual and methodological terms is essential. For that, it is important to mention the strengths and limitations of this research. Although qualitative studies do not aim to establish absolute truth or make statistical inferences about a population, the results provide a partial perspective on the phenomenon, considering the study’s objectives and methodological strategies. This research included only the perspective of patients receiving dental care services and, therefore, it would be important to expand the study to include other key actors in the healthcare system, such as service providers, decision-makers, patients’ families, and civil society organizations. Additionally, examining the experiences of other socially vulnerable groups—such as gender-diverse individuals, internal and international migrants, people with disabilities, rural populations, those affected by internal conflict and violence, and Indigenous communities, among others—would help identify specific needs and barriers that impact their access to and experience of oral healthcare.

An overrepresentation of female participants could be noted. Nevertheless, as mentioned in specific studies, women use dental care more frequently when compared with men. Possible interviewees’ biases were mitigated through triangulation and critical reflexivity.

It is also important to note that qualitative studies are not exempt from potential social desirability bias, in which participants may respond in ways they believe are acceptable to the interviewer or socially approved, limiting the opportunity to express their thoughts or experiences more honestly. To mitigate this, the first author, aware of his role as a healthcare professional, applied principles of critical reflexivity, maintaining constant awareness of their role, social position, values, and personal assumptions. Efforts were made to reduce power asymmetries in the researcher–participant relationship by fostering a climate of trust, avoiding value judgments, and using accessible, non-technical language. This approach contributed to a more accurate interpretation of the results in line with the study’s methodological framework.

The researchers did not use any specialized software; instead, they opted for a manual, hands-on coding approach, utilizing alternative tools such as Microsoft Word and Excel for Windows. This decision does not compromise the quality of the results; on the contrary, it allows for a deeper and more immersive interaction between the research team and the interview transcripts, facilitating continuous interpretive reflection, consistent with the constructivist approach.

However, highlighting the significance of qualitative research in dental practice is important, as the findings of this study allowed for the voices of those directly involved in oral healthcare—namely, the patients—to be heard. Likewise, the GT approach enabled a detailed analysis of the experiences and perceptions of the interviewees, facilitating the creation of a conceptual framework with an interpretative and comprehensive scope. This framework helps to understand the factors perceived as contributors to inequality and inequity in healthcare, which ultimately impact patient safety and dental practice. Most studies on this topic tend to be quantitative [67,68].

### 4.4. Scope and Research Recommendations Derived of This Study

In discussing the study findings, analyzing the current knowledge on patient safety in dentistry is essential. A recent scoping review aimed to synthesize the available evidence on the use of checklists to assess the frequency of adverse events in dental practice [67]. These checklists have been implemented in dental practice as a way to improve dental care quality, preventing errors in oral health services. The main results of this study indicate significant knowledge gaps regarding the implementation of these checklists to enhance safety in dental care. These findings align with those of an integrative review in 2021, which analyzed 91 articles, including narrative reviews, quantitative studies, and qualitative research [68]. It is interesting to mention that only eight studies (8.8%) used qualitative design. This review framed the issue around key research areas such as the frequency of adverse events, primary causes, and measures for mitigation and prevention. However, it highlighted a lack of substantial research on the impact of promotion and prevention strategies in improving patient safety.

Continuing to research topics related to patient safety in dental practice is crucial. Since qualitative research is essential for understanding certain situations or phenomena in health that go beyond statistics or epidemiology, such study designs should be strengthened by involving various key actors who were not included in the present study. Future studies should explore comparisons between public and private services. Additionally, participatory action research should be incorporated to develop co-managed strategies in collaboration between patients (including rural communities and ethnic groups), and key actors within the health system.

## 5. Conclusions

Othering (marginalization/discrimination), inequalities, and inequities are contributing factors that impact any dental care process in terms of patient safety and the quality of dental health services. Therefore, research processes should contribute to the development of strategies based on social realities and operating at different levels of society. It is essential to implement good clinical practices, foster a culture of patient safety, and promote communication and empathy between healthcare personnel and service users. Likewise, the dissemination of knowledge on issues related to oral health inequities, healthcare access, and the safety of dental practice should be encouraged among various actors in civil society, the healthcare system, and policymakers.

Health inequities should be addressed preventively and proactively to be reduced by appropriately combining government policies. This would improve oral health conditions and establish patient safety as a true cornerstone of dental practice, ensuring human dignity and fostering trust in the current healthcare system.

## Figures and Tables

**Table 1:** Sociodemographic description of participants.

Interview Code	Age	Gender Recognition	Socioeconomic Stratum *	Marital Status	Employment Condition	Education Level	Perception in Vulnerability Categories	Provenance
Int1	49	Female	Medium	Single	Employee in General Services (Domestic)	Technical studies	Woman and internal migrant	Rural
Int2	53	Female	Medium	Married	Employee in General Services (Domestic)	Technical studies	Woman, Black woman (Afro-Colombian)	Urban
Int3	40	Female	Medium	Married	Employed as Warehouse Assistant	University	She does not perceive herself as vulnerable	Urban
Int4	43	Male	Low	Single	Administrative employee and union member	Technical studies	Homosexual, he does not perceive himself in categories of vulnerability	Rural
Int5	33	Male	Medium	Single	Executive Director (Service Provision Contract)	Postgraduate studies	Homosexual, HIV positive, he does not perceive himself in categories of vulnerability	Urban
Int6	52	Male	High	Married	Employed as a Publicist	Postgraduate studies	He does not perceive herself as vulnerable	Rural
Int7	23	Female	Medium	Single	University Student	University	She does not perceive herself as vulnerable	Urban
Int8	45	Female	Low	Single	Recycler	No studies	Black woman (Afro-Colombian), single mother, displaced by violence	Rural
Int9	31	Female	Medium	Single	Unemployed	Technical studies	Single mother	Urban
Int10	53	Female	High	Married	Independent advisor	University	She does not perceive herself as vulnerable	Rural
Int11	26	Male	Low	Single	University Student	University	He does not perceive herself as vulnerable	Urban
Int12	52	Female	Medium	Married	Employed as Laboratory Assistant	Technical studies	She does not perceive herself as vulnerable	Urban
Int13	37	Female	Medium	Single	E-commerce	University	Black woman (Afro-Colombian), she does not perceive herself as vulnerable	Rural

* Socioeconomic strata were defined according to Colombia’s official classification by the National Administrative Department of Statistics (DANE, acronym in Spanish), which categorizes households from 1 (very low) to 6 (high) [44].

**Table 2:** Topics addressed during the interview process.

Themes	Axes of Analysis
Vulnerable population	Type of social group: Afro-descendants, people belonging to national, ethnic, religious, or linguistic minorities, migrants and internally displaced persons, people living in extreme poverty, women, children, LGBTIQA+
	Previous expectations and current situation
	Personal and professional future prospects
Labor and academic history	Academic Experience
	Current labor situation
	Labor risks
	Associationism
Patient Safety	Perception/concept/definition of patient safety
	Differences related to the condition of being a vulnerable population
	Perception about the healthcare process
Health situation and conditionings	Self-perception about his/her physical, mental, and oral health status and the health determinants/conditionings
	Perception of the dentist–patient relationship
	Access to health/oral health services
	Perception of the quality of healthcare according to their recognition of being a vulnerable population
Expectations and future	Expectations Vs. Reality in Colombia
	Point of view about the future (short, medium, and long term)

**Table 3:** Microanalysis and categorization of information.

N°	General Categorization/Codification	n	Descriptive Categories
1	Discrimination	20	Discrimination based on vulnerability conditions in oral healthcare
2	Health inequalities	11	
3	Gender approach	10	
4	Health inequities	6	
5	Racism	6	
6	McCarthyism of women	2	
7	Stigmatization of women	1	
8	Suffering from adverse events	19	Suffering from damage or injuries in dental care
9	Sense of vulnerability	8	
10	Therapeutic distancing	6	
11	Failures in care	6	
12	Risk perception	6	
13	Poor quality	6	
14	Inadequate communication	20	Othering and humanization
15	Otherness	15	
16	Humanization of care	7	
17	Empathy	1	
18	Socioeconomic gradient	26	Socioeconomic gradient
19	Access conditions	7	
20	Aesthetics and cosmetics	7	
21	Lack of continuity	2	
22	Payment capacity	1	
23	Therapeutic distrust	12	Oppression and domination by the oral health professional
24	Time limitations	10	
25	Professional supremacy	10	
26	Unethical	2	
27	Oppression-domination by the oral health professional	1	
28	Incompetence	1	
29	Self-care in health	6	Trust generation
30	Satisfaction	5	
31	Trust generation	4	
32	Adherence to biosecurity	3	
33	Therapeutic support	1	Patient empowerment
34	Patient empowerment	4	
35	Enforceability of rights	2	
36	Skepticism	1	
37	Resignation	1	

## Data Availability

Considering the sensitivity of the qualitative data collected during the information gathering process, data is unavailable due to privacy or ethical restrictions. The main discourses from the participants’ perspective are provided in this manuscript.

## Supplementary Materials

The following supporting information can be downloaded at: https://www.mdpi.com/article/10.3390/ijerph22081248/s1. File S1. Interview script (topic guide).
