# Supplementary material for: Generators of Inequality and Inequity Affecting Dental Patient Safety: A Grounded Theory Approach
Source: Int J Environ Res Public Health. 2025 Aug 9;22(8):1248. doi: 10.3390/ijerph22081248 (PMC12386020; doi:10.3390/ijerph22081248)
Supplement: Supplementary file 1 [file ijerph-22-01248-s001.zip › ijerph-3774285-supplementary.pdf]

File S1. Interview script (topic guide)

Phase 1.

General objective:

Understand the factors that generate inequality and inequity in oral health care that affect patient safety from their perspective (the patients).

Specific objectives:

- To describe the processes carried out by patients when using oral health services.
- To reveal the factors that influence the use of oral health services, which lead to inequality and inequity in care and affect patient safety.
- To interpret perceptions of patient safety in the field of oral health care from the users' perspective.
- To recognize specific situations that affect patient safety in oral health care and that cause inequality and inequity in the process.

Elements to consider for the development of interviews:

- Greeting, disposition in a comfortable environment to generate trust
- Introduce the interviewer in a friendly, pleasant manner, without over-emphasizing the situation, and explain the objective of the meeting and the conditions under which the conversation will take place. Explain that it will be recorded, transcribed, and then deleted for the project.
- Implementation of the informed consent procedure, after explaining the project, its potential implications for society, the health system, and dental practice in general, and the specific conditions of your participation as an interviewee in terms of your duties and rights, emphasizing respect for your autonomy and confidentiality in the use and handling of information.
- Sociodemographic aspects of the interviewee

**Interviewee Code**	
**Age**	
**Gender self-recognition**	
**Socioeconomic stratum**	
**Marital status**	
**Condition of employability**	
**Level of education**	
**Conditions of vulnerability**	
**Origin (Rural – Urban)**	

Main questions

1) Have you received dental care in recent years?
2) If the previous answer is affirmative, where did you receive that care, and in what place were you treated?
3) If the previous answer is negative, what is the reason or motive you have not gone into dentistry?
4) Why did you seek dental care?
5) How do you consider the process of getting your dental appointment (quick or slow, close or far, etc.)?
6) How was that last attention you received?
7) Was the situation you asked about resolved at the time?
8) If I asked you to analyze the dental work you had done today, what would your evaluation be?
9) How did you feel during that care?
10) Do you think that, due to your characteristics, for example, age, skin color, or general features, you received different care than other people? Based on your answer, please ask for more information.
11) Did you feel good throughout that process?
12) Was the person who attended to you an assistant or a professional?
13) Was the person who served you a man or a woman?
14) Did the person who attended to you explain, inform you, or discuss with you everything related to your oral condition and what they were going to do to you?
15) During that care, did you feel that they were doing something without your authorization?
16) Do you think patients can be harmed during dental care? Based on the answer, please request an extension.
17) During the care you received, were you able to tell that the person caring for you did things to keep you from feeling bad or unwell? And were you able to tell that they were trying not to harm you?
18) Did you receive an explanation of the bad things that could happen with your treatment and with dental care in general?
19) Were the appointments you were given in a row, or did they take a long time to get?
20) What was the place like, the instruments and equipment with which you were treated?
21) Do you know if all the appointments you were seen for were written in your medical history?
22) Have you ever felt any kind of rejection, mistreatment, or discomfort in the place where you were treated, not only by the professionals but also by other patients?
23) Have you ever felt that the professional made a mistake when receiving care?
24) Have you heard from other dental patients about situations that have affected them?

Based on the answer, please ask for more information.

25) Do you think there is any difference in the care given to people with different situations, such as between the poor and the rich, the educated and the uneducated, men, women, or LGBTI, based on skin color, etc.?

Ask if the interviewee has anything else to contribute or expand on in the conversation. If the answer is affirmative, allow them to express themselves. Otherwise, thank them for their participation in the exercise and reiterate that this information will be used for strict academic purposes.

Phase 2.

General objective:

Understand the factors that generate inequality and inequity in oral health care that affect patient safety from their perspective (the patients).

Specific objectives:

- To describe the processes carried out by patients when using oral health services.
- To reveal the factors that influence the use of oral health services, which lead to inequality and inequity in care and affect patient safety.
- To interpret perceptions of patient safety in the field of oral health care from the users' perspective.
- To recognize specific situations that affect patient safety in oral health care and that cause inequality and inequity in the process.

Elements to consider for the development of interviews:

- Greeting, disposition in a comfortable environment to generate trust
- Introduce the interviewer in a friendly, pleasant manner, without over-emphasizing the situation, and explain the objective of the meeting and the conditions under which the conversation will take place. Explain that it will be recorded, transcribed, and then deleted for the project.
- Implementation of the informed consent procedure, after explaining the project, its potential implications for society, the health system, and dental practice in general, and the specific conditions of your participation as an interviewee in terms of your duties and rights, emphasizing respect for your autonomy and confidentiality in the use and handling of information.
- Sociodemographic aspects of the interviewee

**Interviewee Code**	
**Age**	
**Gender self-recognition**	
**Socioeconomic stratum**	
**Marital status**	
**Condition of employability**	
**Level of education**	
**Conditions of vulnerability**	
**Origin (Rural – Urban)**	

Main questions

1) Have you received dental care in recent years?
2) How can patients be treated in dentistry, according to a scale of economic income or payment possibilities and the quality provided to them, that is, how does the quality a patient receives relate to their economic or payment capacity?
3) What do you think about the possibility of damage or injury caused during dental care when the dentist is arrogant, does not dedicate enough time to care, or considers the patient “less” than he or she is?
4) How can a patient who knows their rights and their health status influence their efforts to avoid discrimination in dental care and avoid oppression and domination by dental professionals?
5) What do you think about the fact that dental professionals who do not understand the patient's suffering, who lack empathy, or who do not care about others, can cause failures in dental care?
6) Do you think there is any difference in the quality and safety of dental care depending on whether the professional is a woman or a man, and can there be more or fewer risks depending on gender?
7) What differences might exist in the quality of dental care between young, white, wealthy, well-educated adults and older, poor, indigenous, or black adults with no education, and in which group does the professional try as much as possible to ensure that there are no gaps in care? Based on the answer, please expand.
8) What are the differences between the dental care offered by private health insurance companies and private dentistry? Which of these offers safer care, and why?
9) What do you think about people who complain about being hurt or having suffered poor dental procedures? Do they receive any kind of punishment from those providing the services?
10) Why might some dentists feel they have power over their patients and can select which patients they care for with more or less confidence?
11) Have you ever experienced, or know of, a case where, due to the treatment you received from a dental professional or due to the occurrence of lapses in care, you felt you did not want to return to care, or you did not want to continue receiving care and preferred to abandon treatment? If the answer is yes, please request an extension.

Ask if the interviewee has anything else to contribute or expand on in the conversation. If the answer is affirmative, allow them to express themselves. Otherwise, thank them for their participation in the exercise and reiterate that this information will be used for strict academic purposes.
